# Brainstem neural mechanisms controlling locomotion with special reference to basal vertebrates

**DOI:** 10.3389/fncir.2023.910207

**Published:** 2023-03-30

**Authors:** Philippe Lacroix-Ouellette, Réjean Dubuc

**Affiliations:** ^1^Department of Neurosciences, Université de Montréal, Montréal, QC, Canada; ^2^Department of Physical Activity Sciences, Université du Québec à Montréal, Montréal, QC, Canada; ^3^Research Group for Adapted Physical Activity, Université du Québec à Montréal, Montréal, QC, Canada

**Keywords:** locomotion, descending control, mesencephalic locomotor region (MLR), neuromodulation, glutamate, acetylcholine

## Abstract

Over the last 60 years, the basic neural circuitry responsible for the supraspinal control of locomotion has progressively been uncovered. Initially, significant progress was made in identifying the different supraspinal structures controlling locomotion in mammals as well as some of the underlying mechanisms. It became clear, however, that the complexity of the mammalian central nervous system (CNS) prevented researchers from characterizing the detailed cellular mechanisms involved and that animal models with a simpler nervous system were needed. Basal vertebrate species such as lampreys, xenopus embryos, and zebrafish became models of choice. More recently, optogenetic approaches have considerably revived interest in mammalian models. The mesencephalic locomotor region (MLR) is an important brainstem region known to control locomotion in all vertebrate species examined to date. It controls locomotion through intermediary cells in the hindbrain, the reticulospinal neurons (RSNs). The MLR comprises populations of cholinergic and glutamatergic neurons and their specific contribution to the control of locomotion is not fully resolved yet. Moreover, the downward projections from the MLR to RSNs is still not fully understood. Reporting on discoveries made in different animal models, this review article focuses on the MLR, its projections to RSNs, and the contribution of these neural elements to the control of locomotion. Excellent and detailed reviews on the brainstem control of locomotion have been recently published with emphasis on mammalian species. The present review article focuses on findings made in basal vertebrates such as the lamprey, to help direct new research in mammals, including humans.

## 1. Introduction

Locomotion is a basic motor act that allows animals to move in their surroundings to generate important functions such as fleeing danger, attacking prey, acquiring food, finding mates, etc. Even though multiple modes of locomotion exist in the different vertebrate animal species (walking, running, flying, or swimming), the neural organization and the mechanisms underlying locomotion are extraordinarily similar (for reviews see Grillner and Wallén, 1985; Grillner and Dubuc, 1988; Grillner and El Manira, 2020; Grillner, 2021; Grillner and Kozlov, 2021). One important discovery made in the 1970s by a group of researchers headed by Sten Grillner was that the spinal cord comprises oscillatory neural units called central pattern generators (CPGs) that generate stereotyped rhythmic activity relying on intrinsic cellular properties and specific connectivity of the neurons involved (Grillner and Zangger, 1975, 1979; for reviews see Grillner, 1985; Kiehn, 2006, 2016; Kiehn et al., 2010; Grillner and El Manira, 2020). The spinal CPGs produce alternating activity in motoneurons controlling flexor and extensor muscles. They are motoneurons controlling muscles that act on more than one joint. Interestingly, these motoneurons were shown to receive excitation during both flexor and extensor phases of the locomotor cycle (Perret and Cabelguen, 1980). Locomotor activity can be recorded directly from muscles of moving animals, but it can also be recorded from spinal ventral roots or peripheral nerves under experimental conditions in which muscles are prevented from contracting with the use of paralyzing agents *in vivo* or by extracting the spinal cord from the animal and maintaining it *in vitro* (Grillner and Zangger, 1975, 1979; Kudo and Yamada, 1987; Smith and Feldman, 1987). This output is referred to as fictive rather than active locomotion because there is no movements produced by the animal. Fictive preparations are particularly useful to precisely establish the activity of different populations of neurons in the central nervous system (CNS) while the circuitry underlying locomotion is active. The absence of movement under these circumstances constitutes a clear advantage for stable recordings. Sten Grillner proposed that there are distinct CPGs controlling each limb segment that are interconnected with one another to coordinate the locomotor movements (Grillner, 1981, 1985; see also McCrea and Rybak, 2008; Frigon, 2012; Kiehn, 2016). This concept has been recently readdressed in further details in a review article (Grillner and Kozlov, 2021).

**FIGURE 1:**
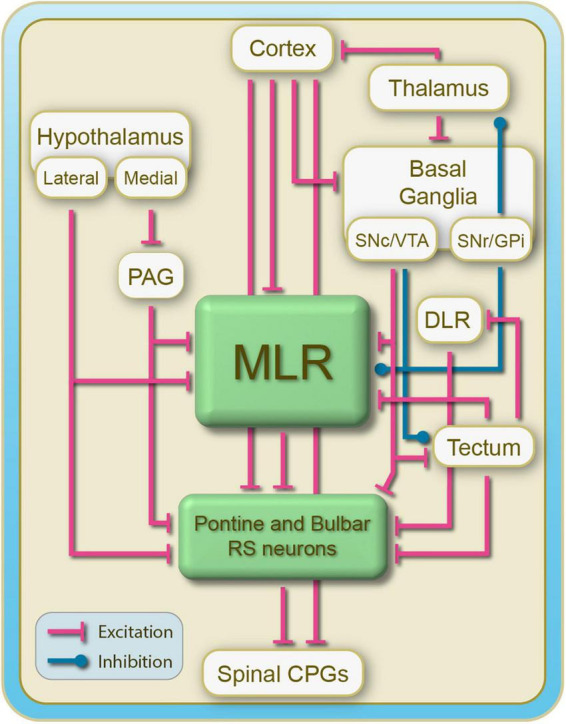
Schematics illustrating the general organization of supraspinal structures controlling locomotion in vertebrates. Reticulospinal cells constitute a large part of the locomotor-related descending inputs that activate the spinal locomotor CPGs. The MLR receives inputs from the basal ganglia and projects extensively to reticulospinal cells. The cortex projects down to the basal ganglia, the MLR and reticulospinal cells. The basal ganglia and the hypothalamus project down to the MLR as well as to reticulospinal neurons. There is another locomotor region, the DLR, that was identified in several species of vertebrates. Far less is known about this locomotor region that projects directly to reticulospinal neurons.

As indicated above, the neural structures located above the spinal cord play a crucial role in controlling the spinal CPGs. Supraspinal neurons are involved in starting, maintaining, and stopping locomotion. In addition, they control changes in speed and direction. How this is achieved is still not fully understood, but it is the focus of ongoing studies in several laboratories around the world. There are many interconnected regions from the forebrain and lower brainstem that contribute to the descending locomotor control. This review focuses primarily on the brainstem regions. Figure 1 provides a general overview of the interconnectivity of CNS regions that play a key role in the control of locomotion in vertebrates.

Although descending control is critical, sensory inputs play a crucial role in adapting locomotor activity to conditions prevailing in the external and internal environments of the animal (not illustrated in Figure 1). These inputs act both at spinal and supraspinal levels (Rossignol et al., 2006; for recent reviews see Akay, 2020; Akay and Murray, 2021).

Locomotion can be elicited by sensory inputs or by internal cues, giving rise to sensory-evoked and goal-directed locomotion, respectively. Visual, auditory, mechanical, or olfactory inputs have all been shown to trigger locomotion (for review see Rossignol et al., 2006). The cellular mechanisms involved in the transformation of olfactory (Derjean et al., 2010; Daghfous et al., 2018; reviewed in Beauséjour et al., 2022), mechanical (Viana Di Prisco et al., 1997, 2000), visual (Suzuki et al., 2019; Isa et al., 2021), and vestibular (Zelenin et al., 2007) inputs into locomotor activity have been identified in lampreys. In this animal species, reticulospinal neurons (RSNs) play a crucial role in relaying sensory inputs to the spinal CPGs for locomotion (Rovainen, 1967a).

The neural mechanisms underlying goal-directed and sensory-evoked locomotion are quite similar with regards to the brainstem and spinal cord. Descending inputs that activate the spinal CPGs originate mostly from RSNs in the pons and the medulla oblongata, which receive sensory inputs from all modalities and ascending inputs from the spinal CPGs. This confers to these cells a role as command cells, a concept that was originally developed for invertebrates (Atwood and Wiersma, 1967). In lampreys, sensory inputs reach RSNs *via* two synapses, olfaction being the exception with at least three synapses required. Whether these almost direct connexions from sensory inputs to RSNs are sufficient for sensory-evoked locomotion remains to be determined. *In vivo* experiments that would examine sensory-evoked locomotion in the absence of CNS regions above the RSNs have yet to be conducted.

As indicated above, goal-directed locomotion is triggered by internal cues associated with basic needs such as food seeking, mate finding or exploration. Studies in mammals have revealed that RSNs located in the hindbrain reticular formation are also involved (Shefchyk et al., 1984; Jell et al., 1985; Takakusaki, 2013) in activating the spinal cord locomotor networks. These RSNs are themselves controlled by upstream locomotor centers. One such center, the mesencephalic locomotor region (MLR), which was discovered around 60 years ago by Russian scientists, is located at the border between the midbrain and the hindbrain (Shik et al., 1966). It was shown that unilateral or bilateral electrical micro-stimulation of this region elicits bilaterally coordinated walking in decerebrated cats. The locomotor output could be controlled by modifying the stimulation intensity, such that increases in stimulation strength allow for the transition between locomotor patterns such as walking, trotting, and galloping. At the time, the MLR discovery revolutionized our understanding of the supraspinal control of locomotion, so much so that scientists began to look for its presence in other species and found it in monkeys (Eidelberg et al., 1981), rats (Skinner and Garcia-Rill, 1984), guinea pigs (Marlinsky and Voitenko, 1991), mice (Caggiano et al., 2018), pigs (Chang et al., 2021), rabbits (Musienko et al., 2008), geese (Sholomenko et al., 1991a,b), salamanders (Cabelguen et al., 2003), stingrays (Bernau et al., 1991), and lampreys (Sirota et al., 2000). Very recently, the MLR was identified in the zebrafish (Carbo-Tano et al., 2022) further supporting the fundamental importance of this structure for locomotor control (see below).

The locomotor-inducing effects of the MLR are relayed to the spinal cord through direct projections to RSNs, as first shown in the cat using intracellular recordings (Orlovskii, 1970; Orlovsky, 1970). Jordan’s group described extensive MLR projections to the bulbar reticular formation (Noga et al., 2003), and showed that bulbar RSNs were necessary for eliciting locomotion by electrical (Jordan et al., 2008) or chemical stimulation (Noga et al., 1988) of the MLR. Similar projections were also shown to be present in the other species into which the MLR was identified (Brocard and Dubuc, 2003; Bretzner and Brownstone, 2013; Ryczko et al., 2016a; Carbo-Tano et al., 2022).

The current understanding relative to the detailed neural circuitry underlying the MLR effects is only partial in mammals. On the other hand, the downstream projections from the MLR to RSNs and the synaptic connectivity of this pathway have been characterized in more details in lampreys. However, this vertebrate model does not allow researchers to genetically manipulate neurons and thus the contribution of specific neuronal populations through their activation or inactivation cannot be defined. Optogenetics experiments are now carried out in species amenable to genetic manipulation to build on previous findings and allow for new questions to be posed. Major advances have been made in understanding the specific contributions of different MLR neural populations (see below) and these findings suggest that the MLR is a far more complex structure than previously thought. The cellular mechanisms underlying the connectivity of descending inputs from the MLR to RSNs have not yet been defined in mammalian models. This is where basal vertebrate models become highly useful. This review reports on the MLR function, its different cell populations, and its downstream connectivity.

## 2. The organization of the MLR in mammals

**FIGURE 2:**
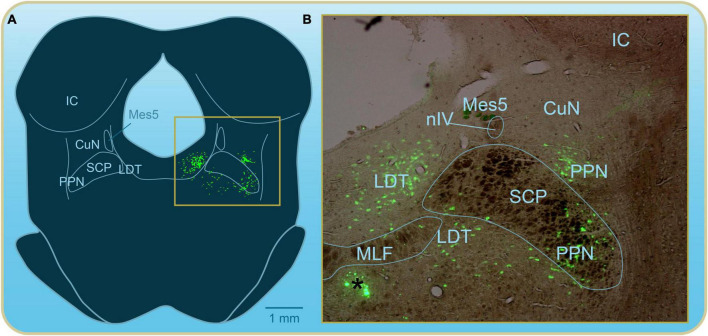
Mesopontine cholinergic cell populations have been described in the brainstem of mammals many decades ago. Here in the rat, much like in lampreys, the cells are arranged in a nearly continuous fashion but have been grouped into nuclei by neuroanatomists for simplicity. The exact names given to each of these nuclei somewhat vary from one species of mammal to the next. The CuN and PPN are traditionally associated with the MLR. **(A)** Schematic representation of a cross section of the isthmic region from the rat brainstem showing the mesopontine cholinergic cell populations (green) in reference to other landmarks in the region. **(B)** Overlay of brightfield and epifluorescence photomicrographs illustrating the cholinergic cells area labeled by immunofluorescence against the enzyme choline acetyltransferase. A black asterisk in the left bottom part of the illustration indicates immunofluorescence artifacts. The frame in panel **(A)** corresponds to the photographed area. CuN, cuneiform nucleus; IC, inferior colliculus; LDT, laterodorsal tegmental nucleus; Mes5, mesencephalic root of the trigeminal nerve; MLF, medial longitudinal fasciculus; nIV, trochlear nerve; SCP, superior cerebellar peduncle.

In this section, we review the anatomical organization of the MLR followed by some of the early findings relative to the role of the different MLR nuclei in the control of locomotion. Discovered in cats, the MLR was first defined physiologically as a brainstem region from which it is possible to elicit coordinated and controllable locomotion. The amplitude of the muscle bursts and their frequency changed as the experimenters varied the intensity or frequency of stimulation. As such, the MLR was proposed to control the power of locomotion (Shik et al., 1966; Sirota et al., 2000), somewhat like a rheostat. In the original cat experiments, the MLR was found to be located at the junction between the midbrain and hindbrain, in the cuneiform nucleus (CuN; Shik et al., 1966). Later studies by the group of Edgar Garcia-Rill showed that the pedunculopontine nucleus in rats (PPN; Figure 2) was the major contributor in the MLR effects (Garcia-Rill et al., 1987). They found a striking similarity between the location of the efficient stimulation sites and that of cholinergic neurons in the PPN. This study and several others generated some confusion as to the exact anatomical structures that constituted the MLR. For many years, it was recognized that both the CuN and the PPN were part of the MLR (Figure 2). More recent studies in basal vertebrates have revealed that the area around another cholinergic nucleus located somewhat more caudally and medially, the laterodorsal tegmental nucleus (LDT), was highly efficient in eliciting locomotor activity and likely to correspond to the MLR in these species (Cabelguen et al., 2003; Le Ray et al., 2003, 2011). Whether the contribution of the area around the LDT is a specific feature of basal vertebrate species remains to be determined.

We will now describe the organization of the CuN and the PPN in mammals and their first uncovered contributions to the control of locomotion. We will then describe the organization of the MLR in lampreys.

### 2.1. The cuneiform nucleus

In humans, the CuN has been anatomically defined by its location and cell morphologies (Olszewski and Baxter, 1954). It is ventral to the inferior and superior colliculi in the dorsolateral part of the mesencephalic tegmentum. In the cat, the CuN is also ventrally located to both the superior and inferior colliculi (Taber, 1961; Edwards, 1975). The rat CuN is described as a midbrain reticular structure spreading from the rostral pons to the pretectal thalamus (Zemlan and Behbehani, 1984, 1988).

The CuN contains GABAergic neurons in cats (Appell and Behan, 1990; Pose et al., 2000) and rats (Ford et al., 1995). Glutamatergic neurons have been observed in the rat (Heise and Mitrofanis, 2006) in support of previous studies describing glutamate efferent projections from the CuN to surrounding structures (Beitz, 1989; Beart et al., 1990). A small number of cholinergic cells have been observed in the rat (Spann and Grofova, 1992), where they were viewed as “misplaced” satellite cells from the PPN (Noback, 1959). Only a small number of cholinergic neurons were identified near the cat CuN (Kimura et al., 1981; Jones and Beaudet, 1987; Jones, 1991), specifically in the ventral subcuneiform nucleus (subCuN). Cholinergic neurons were found in the subCuN (and PPN) of the monkey, but the authors suggested that the entire region was the PPN area (Smith and Parent, 1984). Remarkably, these studies show that, from rats to humans, the CuN is located in similar areas, bordered by the same structures, and populated by cells with comparable morphologies.

The CuN sends descending projections to many areas of the CNS. It projects to RSNs, a connection crucial for the operation of the MLR in controlling locomotion. The CuN projects to the magnocellular reticular nucleus in cats (Abols and Basbaum, 1981), rats (Beitz, 1982), and monkeys (Chung et al., 1983). A descending pathway from the CuN was described that mainly reached the ipsilateral pons and medulla in the cat (Steeves and Jordan, 1984) and in the rat (Bernard et al., 1989). The cat study reported additional projections to the dorsal tegmental nucleus and nucleus raphe magnus.

Several years after the MLR was identified in several vertebrate species, Sinnamon (1993) proposed that different parts of the MLR were associated with specific behavioral contexts. For instance, he proposed that the CuN was involved in defensive responses (for review, see Grillner et al., 1997; Jordan, 1998). Because the CuN was also associated with cardiovascular function and analgesic responses (Korte et al., 1992; Lam et al., 1996), it was linked to life threatening situations such as fleeing from a predator, allowing physiological adjustments to facilitate such behaviors. Moreover, the CuN was shown to be activated alongside other defense-related structures during social (Kollack-Walker et al., 1997) and predator-associated stress (Dielenberg et al., 2001).

Electrical stimulation of the CuN triggers aversive or escape responses in freely moving cats (Mori et al., 1989) and rats (Depoortere et al., 1990; for review, see Grillner et al., 1997). Moreover, micro-injections of glutamate in the rat CuN area causes freezing as a result of a first injection; running occurs with additional ensuing injections (Mitchell et al., 1988a,b; Kafkafi et al., 2003), which suggests that gradual recruitment of more CuN neurons leads to more intense locomotor activity. Although the CuN can elicit aversive or escape behaviors, it also elicits controlled locomotion as initially described by Shik et al. (1966) and subsequent studies. In the freely moving cat, electrical stimulation of the CuN increased the speed at which they were able to cross path in search of food (Sterman and Fairchild, 1966). Stimulation of the CuN was also able to produce rhythmic coordinated movement of limbs in the decerebrated monkey (Eidelberg et al., 1981). Increasing stimulation intensity was associated with an increase in movement frequency, and interestingly, with gait transition from walking to galloping. Jankowska et al. (2011) confirmed that the stimulation of the cat CuN elicited synaptic responses in RSNs. The specific nature of these synaptic responses remains to be established in the mammalian models.

### 2.2. The pedunculopontine nucleus

Studies on the MLR carried out in the 1980s suggested that while the CuN was part of a defensive system, the PPN was part of an exploratory system (for review, see Jordan, 1998). The PPN was first identified using cell morphology criteria established by Olszewski and Baxter (1954). At that time, a cholinergic cell nomenclature was not established. Later, the Ch5 nucleus was reported to overlap with the PPN (Mesulam et al., 1983). At the time, the rat PPN was even defined as a nucleus composed exclusively of large multipolar cholinergic cells (Rye et al., 1987). Later studies revealed that the PPN is heterogenous and not exclusively cholinergic (Mena-Segovia et al., 2009; Wang and Morales, 2009; Martinez-Gonzalez et al., 2012). In addition to cholinergic neurons (Clements and Grant, 1990; Spann and Grofova, 1992; Ford et al., 1995), the PPN contains glutamatergic and GABAergic neurons (Wang and Morales, 2009). The specific role of the different populations of PPN neurons is now actively investigated using optogenetics (see below).

The PPN sends projections to the midbrain, pons, medulla, and spinal cord (for review, see Inglis and Winn, 1995; Martinez-Gonzalez et al., 2011). Although the PPN has also abundant ascending projections to the forebrain (see Caggiano et al., 2018) we have decided to focus on the descending projections. In the rat and cat, cholinergic neurons of the PPN innervate cells across a large portion of the hindbrain reticular formation. Anterograde tracing experiments revealed that the pars compacta of the cat PPN sends descending projections that reach the pontine and bulbar reticular formation (Edley and Graybiel, 1983; Skinner et al., 1990; Inglis and Winn, 1995; Karachi et al., 2010; Martinez-Gonzalez et al., 2011; Mazzone et al., 2012) and 47% of descending axons to the pontine reticular formation are from PPN cholinergic cells (Takakusaki et al., 1996). Projections to the spinal cord have been reported in the rat (Rye et al., 1988; Spann and Grofova, 1989), but not in the cat (Edley and Graybiel, 1983). Other neurotransmitters present in the PPN, namely glutamate, may also have a more important role than formerly thought as will be discussed below in relation to lampreys.

Stimulation of the PPN in rats (Garcia-Rill et al., 1987) was shown to elicit well-coordinated locomotion and the sites where electrical or chemical stimulation most reliably induced coordinated locomotion corresponded to the location of cholinergic cells in the PPN. As shown in the cat (Shik et al., 1966), the intensity of the locomotor output increased with the strength of stimulation. Interestingly, it was shown more recently that the loss of cholinergic neurons in the PPN in humans is associated with locomotor deficits as seen in Parkinson’s disease (Karachi et al., 2010). These findings have spiked the interest in the MLR and its role in gait disorders.

## 3. Descending locomotor control in lampreys

Lampreys have been used for decades to study the neural mechanisms underlying motor control and sensorimotor integration. They are basal vertebrates that have diverged from the main vertebrate lineage more than 550 million years ago and therefore their CNS is considered a “blueprint” of the vertebrate brain (Robertson et al., 2014). Interestingly, the general organization of the lamprey CNS is very similar to that of mammals, but because it contains much fewer neurons, it is more amenable to study cellular mechanisms and behavior simultaneously, an important goal in neuroscience. During his pioneering work on the lamprey, Rovainen (1967a,b) developed an *in vitro* preparation that contributed importantly to characterizing cellular connectivity in the brainstem and spinal cord.

As in other vertebrate species, the lamprey brainstem has both sensory and motor functions, and is also the seat of respiratory and cardiovascular functions. The lamprey and the more recently evolved vertebrates share several neural network features, from pathways to intrinsic neuronal properties (Dubuc et al., 2008). Using the lamprey as a model to characterize the neural mechanisms underlying locomotion has therefore several advantages. The lamprey brain comprises a simpler neural circuitry and its neurons are more easily accessible for recordings, which provides the possibility to define neural activity of single and multiple neurons during fictive and active locomotion (semi-intact preparations). Many key discoveries related to locomotion have been made using the lamprey model and some of them are listed below.

**FIGURE 3:**
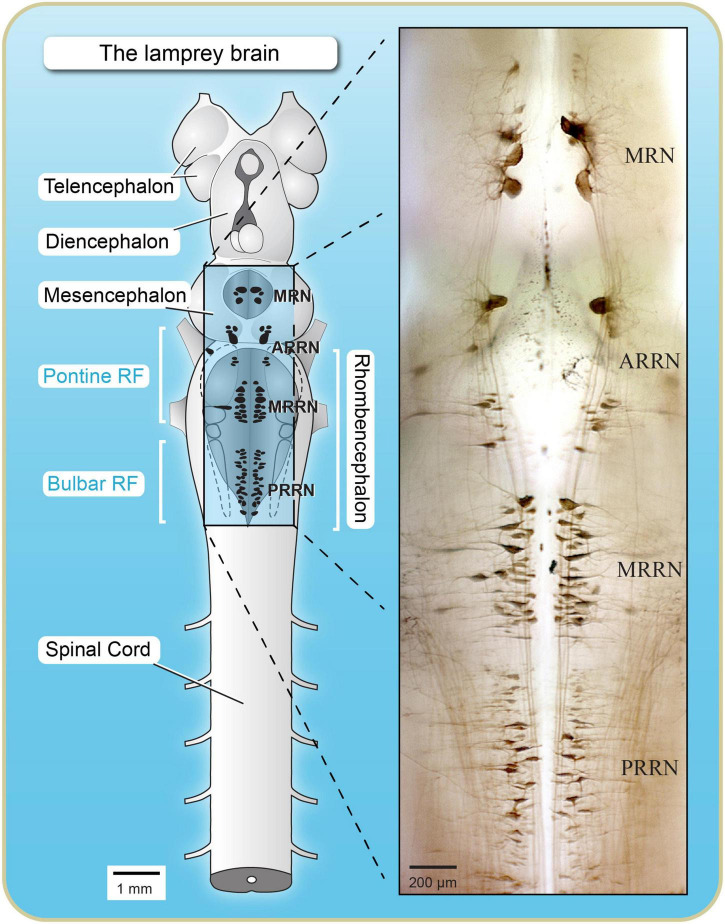
Dorsal view of the brain and rostral spinal cord of lampreys illustrating reticulospinal cells that were labeled by an injection of cobalt-lysine in the rostral spinal cord. **(Left)** Schematics illustrating the location of reticulospinal neurons (dark cells). **(Right)** Brain mounted on a microscope slide (wholemount preparation). Many of the reticulospinal cells are located just below the ventral surface of the fourth ventricle (PRRN, MRRN, and ARRN), making them easily accessible for intracellular/patch recordings/imaging as well as for local injections of drugs. Adapted from Bussières (1994).

Lamprey RSNs provide the link between rostral supraspinal structures and spinal cord neurons to produce movement (Figure 3). The lamprey reticular formation consists of four nuclei: the mesencephalic reticular nucleus (MRN), and three rhombencephalic (pontine and bulbar) reticular nuclei: the anterior (ARRN), the middle (MRRN), and the posterior (PRRN) (Nieuwenhuys, 1972, 1977; Figure 3). There is approximately 2,500 RSNs in lampreys and the PRRN and MRRN together comprise around 90% of these (Bussières, 1994; Figure 3). In the middle of the 19th century, Johannes Müller described large axons in the lamprey spinal cord (Müller, 1840; Rovainen, 1978). The axons were so large that a dozen of them occupied the entire ventromedial quadrant of the spinal cord. The cell bodies of these axons were later identified as located in the reticular formation, and the scientists of the time referred to them as “Müller cells.” Because of their large size and distinct morphologies, the Müller cells attracted considerable attention from microscopists (Johnston, 1902; Tretjakoff, 1909). Studies in other vertebrate species revealed that the organization of the reticular formation of lampreys is very similar to that of other basal vertebrates such as fish and amphibians (Cruce and Newman, 1984).

In the 1960s, Carl Rovainen published several elegant studies defining the physiological characteristics of the Müller cells (for review see Rovainen, 1978). Lamprey RSNs were shown to make direct synaptic connections with spinal motoneurons and interneurons that compose the CPG for locomotion (Buchanan et al., 1987). RSNs constitute the final descending pathway for locomotion. A large proportion of the descending axons use glutamate as their neurotransmitter (Dale, 1986; Dale and Grillner, 1986). It is not surprising therefore that, in lampreys, fictive locomotion can be elicited in the *in vitro* isolated spinal cord by adding only glutamate or NMDA to the perfusing Ringer’s solution (Grillner et al., 1981; Sigvardt et al., 1985; Wallén and Grillner, 1987). In the intact lamprey, RSNs activate the spinal CPGs whether locomotion is elicited by sensory inputs or by the MLR. For sensory-evoked locomotion, the brainstem sensory relay cells activated by sensory inputs directly excite RSNs that in turn send excitatory inputs to spinal interneurons taking part in the generation of locomotion (Dubuc et al., 2008; Ryczko and Dubuc, 2013; Daghfous et al., 2016). The next sections will describe the contribution of RSNs to the control of locomotion elicited by stimulation of the MLR.

### 3.1. The lamprey MLR

The lamprey MLR was first identified at the beginning of the Millennium by the group of Réjean Dubuc (Sirota et al., 2000). Originally, it was shown that electrical stimulation of a region loosely corresponding to the lamprey PPN elicited controllable and well-coordinated swimming in a semi-intact preparation consisting of the brain and the rostral spinal cord (ca. 10 segments) dissected out in vitro, with the caudal part of the body intact and capable of swimming. It was later found that stimulation of a region corresponding to the location of the LDT was more reliable in eliciting controllable and coordinated swimming (Brocard and Dubuc, 2003; Le Ray et al., 2003; Brocard et al., 2010; Smetana et al., 2010; Juvin et al., 2016; Grätsch et al., 2019). Interestingly, the location of the MLR in salamanders also coincides with cholinergic cells of the LDT in the isthmic region (Cabelguen et al., 2003; Ryczko et al., 2016a).

**FIGURE 4:**
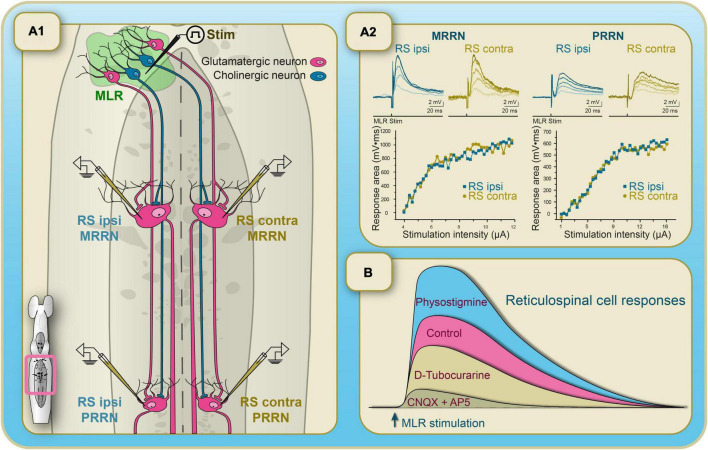
A unilateral stimulation of the MLR in lampreys elicits coordinated and symmetrical swimming movements on both sides. Physiological experiments showed that the MLR on one side of the brainstem provides highly symmetrical glutamatergic and cholinergic inputs to paired, giant RS cells from opposite sides of the brainstem. Anatomical experiments confirm the presence of bilateral projections from the MLR to RS cells. **(A1)** Diagram of the hindbrain showing the bilateral connections between the MLR and paired, giant RS cells as well as the position of the electrodes for the electrophysiological experiments. **(A2)** Left side: the MLR on one side is stimulated at increasing intensities and the synaptic responses are recorded in paired, giant RS cells located in de pontine reticular formation (MRRN) on opposite sides of the brainstem. Note that the responses are remarkably similar on both sides. Right side: Same as on the left side, but for paired large RS cells in the bulbar reticular formation (PRRN). **(B)** Schematized illustration of the subthreshold synaptic responses elicited in reticulospinal neurons by stimulation of the MLR. Glutamatergic and cholinergic transmission were blocked or potentiated sequentially by adding different pharmacological agents. The EPSPs were increased under physostigmine, an inhibitor of the re-uptake of acetylcholine. The EPSPs were significantly decreased under D-tubocurarine, a nicotinic receptor antagonist, and were further decreased by the addition of a mixture of CNQX/AP5, glutamatergic receptors antagonists. A small response from unknown origin remained. Adapted from Brocard et al. (2010).

The initial study describing the MLR of lampreys clearly established that the inputs from the MLR were relayed by hindbrain RSNs before reaching the spinal cord to produce swimming. The MLR inputs to hindbrain RSNs were then examined in detail. Two reticular nuclei are in the rostral half of the lamprey hindbrain, a region homologous to the pons of mammals, namely the anterior (ARRN) and middle (MRRN) rhombencephalic reticular nuclei, and one large reticular nucleus is in the caudal half, homologous to the medulla oblongata, namely the posterior (PRRN) rhombencephalic reticular nucleus (Cruce and Newman, 1984; Figure 3). The contribution of the pontine and bulbar reticular formation to the control of locomotion in lampreys was examined in electrophysiological experiments (Brocard and Dubuc, 2003). The MLR was stimulated electrically while synaptic responses were recorded from RSNs (Figure 4A1). Dual intracellular recordings of RSNs were also carried out to compare synaptic responses in different parts of the reticular formation as well as responses from both sides of the brainstem. Following MLR stimulation, RSNs in the bulbar PRRN displayed synaptic responses occurring at a longer latency as compared to RSNs in the pontine MRRN (Figure 4A2). The longer latencies resulted from the PRRN being at a greater distance from the MLR than the MRRN. The stimulus/response relationships were similar for RSNs in both nuclei, with the same threshold intensity that evoked synaptic response (Figure 4A2). On the other hand, the MLR-evoked responses had a greater magnitude in pontine MRRN RSNs than in bulbar PRRN RSNs. It was argued that intrinsic properties of RSNs were not responsible for this difference because larger responses would have been expected in PRRN cells due to their larger input resistance. It is possible that a larger density of projections onto pontine MRRN neurons compared to bulbar PRRN neurons could explain the difference in synaptic response size. The time to peak of the responses was also shorter in MRRN neurons (Figure 4A2). It was hypothesized that this could be due to a disparity in ionic current kinetics or more synchronous synaptic inputs to MRRN cells (Brocard and Dubuc, 2003). The contribution of the MRRN and PRRN to MLR-induced locomotion was also examined. Following injections of glutamate antagonists in the pontine MRRN, the MLR stimulation threshold for eliciting locomotion was markedly increased. Moreover, in experiments during which locomotor activity was already elicited by stimulating the MLR, the injection of the antagonists in the MRRN suppressed locomotor activity (Brocard and Dubuc, 2003). Injections of glutamate receptor antagonists in the bulbar PRRN had much weaker effects. These results altogether indicate that projections from MLR to the pontine reticular formation play a crucial role in eliciting locomotion. Most of the studies carried out in mammals have examined MLR inputs to the bulbar and not the pontine reticular formation. If lessons were to be taken from the lamprey model, more emphasis should be given to the pontine reticular formation.

**FIGURE 5:**
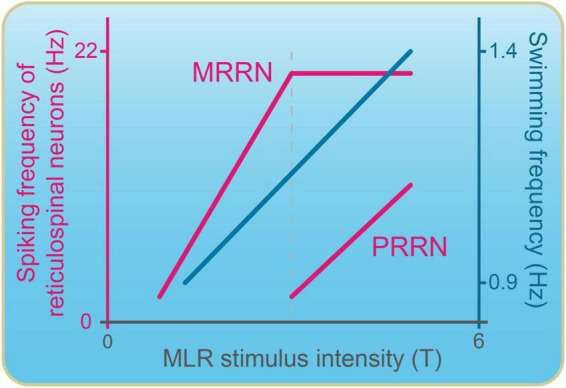
Recruitment pattern of reticulospinal neurons as the stimulation in the MLR is increased. The spiking frequency of reticulospinal neurons and swimming frequency are plotted against the intensity of MLR stimulation. Reticulospinal neurons in the pons (MRRN) are recruited at low MLR intensity and rapidly reach a discharge frequency plateau. Bulbar reticulospinal neurons (PRRN) are recruited at higher MLR stimulation intensities. They start discharging when the discharge frequency of pontine reticulospinal cells has reached a plateau. Adapted from Brocard and Dubuc (2003).

The recruitment pattern of pontine MRRN vs. bulbar PRRN RSNs was also examined in the lamprey model (Figure 5). RSNs in the MRRN discharged at lower stimulation intensities compared to cells in the PRRN. The latter began discharging when the RSNs in the MRRN had already reached a maximum level of discharge. It was proposed that bulbar PRRN cells could provide additional excitation to CPG neurons to increase locomotor speed (see also Wannier et al., 1998).

Altogether, the findings above indicate that MLR inputs onto the lamprey reticular formation are not evenly distributed throughout the reticular formation and that different populations of RSNs are likely to contribute differently to the control of locomotion.

### 3.2. Bilaterally symmetrical activation of RSNs

In the animal species into which the MLR was identified as of now, stimulating the MLR on one side systematically resulted in bilaterally symmetrical locomotion. This suggests that the MLR projects to both sides of the hindbrain. The issue of bilateral effects of the MLR was examined in the lamprey where injections of anatomical tracers in the hindbrain reticular formation on one side revealed the presence of retrogradely labeled neurons in the MLR on both sides (Sirota et al., 2000). A few years later, it was shown that there was an ipsilateral bias in the MLR projections as fewer labeled neurons were found in the contralateral MLR (Brocard et al., 2010). On the other hand, the anatomical asymmetry seemed to be compensated physiologically. It was shown that a unilateral MLR stimulation elicits bilaterally symmetrical inputs in RSNs, thus contributing to symmetrical locomotion patterns (Figure 4A2; Brocard et al., 2010). The bilateral projections are monosynaptic as demonstrated by bathing the brainstem in a high-divalent cation solution. The synaptic response in RSNs displayed a constant latency during high-frequency stimulations. Furthermore, when normal Ringer’s solution was gradually replaced with a Ca^2+^-free solution, the intensity of responses in RSNs showed gradual reduction (Brocard et al., 2010). These data strongly support monosynaptic connections between the MLR and RSNs. In the same study, pharmacological activation of the MLR recruited RSNs in the same way as did electrical stimulation. This suggests that this observed symmetry is produced by cell bodies located around the stimulation electrode, supporting the previous anatomical findings.

### 3.3. Activation patterns in RSNs

Recent studies in the lamprey show that RSNs respond to MLR stimulation with three patterns of activity. Some RSNs display a burst of discharges at the beginning of locomotion, others maintain their discharge throughout the locomotor bout, while a third type show bursts at the beginning and at the end of locomotor activity (Juvin et al., 2016). The three populations of RSNs were named Start, Maintain, and Stop cells, respectively. To determine if the Stop cell population was involved in the termination of locomotion, activation and inactivation experiments were conducted. After initiation of locomotion by MLR stimulation, an injection of D-glutamate over the caudal part of the pontine MRRN activated Stop cells, Start cells and interneurons in that region, but most importantly, it lead to the termination of locomotion. This could be due to a larger number of Stop cells being activated, or Start cells being in a low state of excitability during ongoing locomotion. Again, with ongoing MLR-evoked locomotion, Stop cells were then inactivated using local injections of the glutamatergic antagonists, CNQX/AP5. In those experiments, the duration of the locomotor activity was not significantly modified, but the length of the deceleration period was extended and less abrupt. The authors suggested that Stop cells are probably not the only cells providing a termination command, but they could be needed in cases where locomotion must stop quickly. The identification of Stop cells in the reticular formation of lampreys followed observations made by the group of Kiehn in mice (Bouvier et al., 2015). They elegantly showed that selective activation of V2a neurons of the rostral medulla stopped ongoing locomotor activity and that inactivation of such neurons decreased spontaneous stopping *in vivo*. Interestingly, the Stop neurons found in lampreys were located in a similar region of the reticular formation, suggesting that the anatomo-physiological features of the locomotor control system are well conserved in vertebrates.

In lampreys, it was later discovered that the stop signals originated in the MLR. High intensity stimulation to the MLR evoked the initiation of locomotion, but then a second lower intensity stimulation terminated the locomotor event (Grätsch et al., 2019). These findings suggested that the MLR can control such opposite behaviors. In the same study, stimulating the MLR while ejecting glutamate agonist over the Stop cell region in the pontine MRRN elicited a decrease in swimming duration, whereas cholinergic agonist showed no change in such component of swimming. To confirm the role of glutamate in the MLR termination command, glutamate receptors were blocked in Stop cells, which prevented the reduction in the duration of the locomotor activity observed after application of the second lower-intensity stimulation. These findings in the lamprey show quite clearly that glutamatergic inputs from the MLR to Stop cells are responsible for terminating locomotor activity, while cholinergic inputs do not seem to be involved in activating Stop cells. Interestingly, it was shown in mammals that activation of GABAergic neurons of the CuN or PPN stops locomotion momentarily (Roseberry et al., 2016; Caggiano et al., 2018).

### 3.4. Neurotransmitters involved in transmitting signals from the MLR to RSNs

In lampreys, many choline acetyltransferase-immunoreactive cells were found in the isthmic region (Pombal et al., 2001). This was confirmed by Le Ray et al. (2003) who also showed that MLR-evoked locomotion relies on both glutamatergic and cholinergic inputs (Figure 4B). The authors showed a dose-dependent receptor-mediated depolarization following local ejections of acetylcholine onto RSNs. This response persisted after adding TTX to the perfusion Ringer’s solution, suggesting a direct effect onto RSNs. Because single injections did not always produce reliable responses, it was argued that a slow build-up was needed following MLR stimulation to produce locomotion that could be the result of nicotinic inputs. As expected, the nicotinic cholinergic antagonist, D-tubocurarine, blocked this build-up. In addition, when locomotion was induced by NMDA perfusion onto the spinal cord, nicotinic activation of RSNs caused an acceleration of the locomotor rhythm. It is noteworthy that a previous study had shown that cholinergic neurotransmission had no effect on RSNs when bath-applied (Matthews and Wickelgren, 1979). The absence of effect in bath-applied conditions may have been due to desensitization of the cholinergic receptors.

Le Ray et al. (2003) suggested that cholinergic inputs to RSNs could cooperate with glutamatergic inputs to induce locomotion. It was found that the two neurotransmitter systems were involved in the synaptic inputs from the MLR to RSNs (Figure 4B). The synaptic responses observed under control conditions were significantly reduced by adding a glutamate antagonist to the perfusing Ringer’s solution (Figure 4B). Additionally blocking nicotinic cholinergic receptors reduced even further the synaptic responses, whereas blocking cholinergic reuptake markedly increased the responses. These findings indicate that glutamate and acetylcholine transmission play a role in the MLR-induced activation of RSNs. How these two neurotransmitter systems interact remains to be determined.

### 3.5. Muscarinic contribution

**FIGURE 6:**
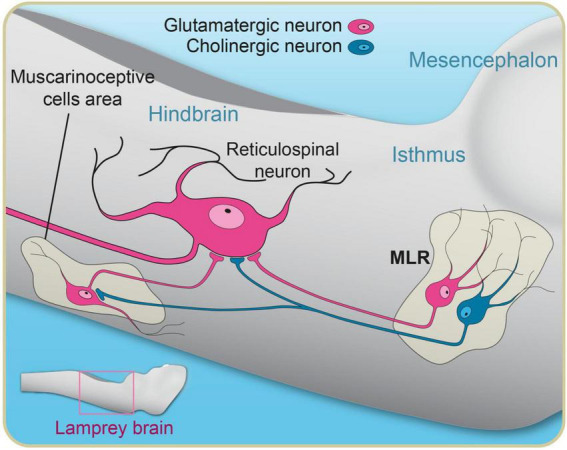
Schematic illustration of downward projections from the MLR to the pontine reticular formation. In addition to direct projections from glutamatergic and cholinergic MLR neurons to reticulospinal neurons, there is a muscarinic cholinergic projection from the MLR to a group of muscarinoceptive cells in the hindbrain that in turn provide extra excitation to reticulospinal neurons. This pathway is believed to boost the locomotor output at high swimming speeds, acting as a hyperdrive mechanism for locomotion. Adapted from Smetana et al. (2010).

Sensory inputs are crucial to the control and modulation of locomotion (for review see Rossignol et al., 2006). In lampreys, cholinergic inputs from the MLR were shown to also modulate sensory transmission. A local application of muscarinic agonists on RSNs reduced their response to a sensory stimulation (Le Ray et al., 2004), whereas application of an antagonist produced the opposite effect. A similar decrease in synaptic sensory transmission to RSNs occurred when the MLR was stimulated (Le Ray et al., 2010). The depression of sensory transmission was correlated to the intensity of stimulation of the MLR. The authors proposed that as the MLR induces locomotion (goal-directed), it reduces sensory inputs that could perturb the locomotor activity (Le Ray et al., 2010). Another role of cholinergic inputs from the MLR was described by Smetana et al. (2010). The authors showed that a parallel cholinergic pathway from the MLR activated downstream muscarinoceptive cells in the hindbrain (Figure 6) and that those cells in turn projected to RSNs to increase their level of excitation. The pathway was recruited only at high levels of activation of the MLR. The authors argued that this pathway was boosting local motor output and they described it as a brainstem hyperdrive mechanism for locomotion (Figure 6).

### 3.6. Salamanders and zebrafish

In tetrapods, the downstream projections from the MLR to brainstem neurons remained unknown until recently. Ryczko et al. (2016b) examined the brainstem circuits from the MLR to identified RSNs in the salamander *Notophthalmus viridescens*. They showed bilateral projections from the MLR to hindbrain RSNs, and calcium-imaging coupled to electrophysiology techniques revealed that a unilateral MLR stimulation produced very similar responses in RSNs on both sides. Bath-application or local microinjections of glutamatergic antagonists markedly reduced RSN responses. The authors showed that the brainstem circuits activated by the MLR in salamanders are organized very similarly to those of lampreys.

In zebrafish, questions relative to the connectivity between the different brain structures involved in the neural control of locomotion have been examined efficiently for many years using combined approaches such as optogenetics, imaging, electrophysiology, and behavior (Thouvenin and Wyart, 2017; Severi et al., 2018). There is overwhelming evidence for a crucial role of RSNs neurons in the descending control of locomotion in zebrafish (see Kimura et al., 2013). However, despite the multiple approaches used, the zebrafish MLR has remained elusive. For instance, stimulation of the nucleus of the medial longitudinal fasciculus (nMLF) elicits locomotion in zebrafish. It was suggested from these experiments that the zebrafish nMLF could correspond to the MLR in these animals (Green and Hale, 2012). However, the anatomical location of the nMLF is far from being homologous to that of the mesopontine border-located MLR in other basal vertebrates. It is likely that the observed stimulation effects in those zebrafish experiments were equivalent to stimulating RSNs as seen in other species. The zebrafish MLR was recently uncovered as a small region dorsal to the locus coeruleus with glutamatergic and cholinergic neurons (Carbo-Tano et al., 2022). Stimulation of this newly-identified zebrafish MLR reliably elicited forward bouts of controlled duration and speed. The same authors showed that locomotion resulted from the activation of V2a RSNs in the pontine and retropontine regions, as well as in the medulla. These recent findings bring the zebrafish in the forefront as a highly useful animal model to characterize the detailed neural mechanisms underlying the supraspinal control of locomotion. Moreover, the strikingly similar location of the zebrafish MLR as compared to that of the lamprey indicates that basal vertebrates use very similar brainstem neural mechanisms to control locomotion.

## 4. Revisiting the mammalian MLR

Research carried out in lampreys has provided new and valuable information on the operation of the MLR and RSNs during locomotion. These findings have in part paved the way for new research in mammalian models. The impressive advances made in mouse genetics have also provided exciting new approaches with optogenetic techniques. We have described above the general organization of the MLR as it stood historically. We will now present some of new findings made in mammals. Excellent recent review articles have been written on this subject (Leiras et al., 2022; Noga and Whelan, 2022). We will first address recent findings on the connectivity between the MLR and RSNs in mammals and then the new findings on the specific role of genetically-identified MLR neurons in locomotor behavior.

Early neuroanatomical studies had shown that the pontine reticular formation receives inputs from the MLR in cats (Garcia-Rill, 1983; Garcia-Rill et al., 1983a,b,c; Steeves and Jordan, 1984) and in rats (Garcia-Rill, 1986; Garcia-Rill et al., 1986). The presence of cholinergic neurons in the MLR suggested that acetylcholine played a role in activating RSNs. It was shown that carbachol, a muscarinic agonist, excited pontine reticular neurons, whereas a muscarinic antagonist blocked PPN-induced excitation in the same neurons. This suggested that the effects were due to muscarinic receptor activation (Homma et al., 2002). On the other hand, carbachol did not elicit locomotion when injected into the avian pontine reticular formation (Sholomenko et al., 1991a,b), contrary to what happens in the rat (Mamiya et al., 2005). Cholinergic agonists injected into the rat ventromedial medulla induced locomotion (Kinjo et al., 1990), an effect that was blocked by a cholinergic antagonist. However, in the avian model, carbachol did elicit locomotion when injected in a region homologous to the cat bulbar reticular formation (Sholomenko et al., 1991a,b). Taken together, these results suggested a key role of PPN cholinergic projections in MLR-induced locomotion. Unfortunately, the cellular mechanisms by which the MLR effects were exerted remained unknown. Moreover, the discrepancies between different species have not been addressed as well as the differential effects of cholinergic inputs on pontine vs. bulbar RSNs.

Few studies have directly characterized the MLR inputs onto RSNs in mammals. In an elegant study, Bretzner and Brownstone (2013) showed that bulbar RSNs expressing Lhx3 and/or Chx10 in mice received inputs from the MLR. Moreover, these neurons displayed an increased expression of c-Fos associated with motor tasks, and their electrophysiological properties suggested that they were indeed involved in the control of locomotion. Glutamatergic neurons within the lateral paragigantocellular nucleus (LPGi) were recently shown to be essential for high-speed locomotion as they receive glutamatergic inputs from the CuN (Capelli et al., 2017).

Specific populations of RSNs are now known to be involved in stopping locomotion. Photo-stimulation of some Chx10 positive neurons in the hindbrain halted ongoing locomotion (Bouvier et al., 2015). This indicated that not all descending RSNs produce excitatory effects in the spinal cord. Interestingly, a population of Stop cells was later described in lampreys at the same location in the reticular formation (Juvin et al., 2016; and see above). The descending inputs to RSNs are likely to be organized in a complex fashion, whereby different inputs may activate, maintain, or stop locomotion. Some other specific inputs may be involved in steering and controlling speed. As indicated above, glutamatergic, and cholinergic neurons are present in the mammalian MLR. The contribution of these two neurotransmitter systems to the activity of RSNs has not yet been fully resolved. On the other hand, the behavioral effects of activating specific populations of glutamatergic and cholinergic neurons in the MLR have been examined in several studies using optogenetic tools in mammals.

A recent study by the group of Ole Kiehn (Caggiano et al., 2018) revealed that glutamatergic subpopulations of neurons in both the PPN and the CuN control slow alternating locomotion. The glutamatergic subpopulations in both the PPN and the CuN would maintain ongoing locomotion in the walk and trot range. The authors suggested that glutamatergic neurons in the PPN would promote locomotion for the purpose of explorative behavior, whereas those in the CuN would promote escape locomotion. These important findings confirm that both the PPN and the CuN show a significant contribution to the control of locomotion, but to seemingly different aspects of the motor output. A study from the group of Frédéric Bretzner revealed that distinct cell populations in the midbrain exhibit phasic or tonic effects to control posture or locomotion (Josset et al., 2018). The authors showed that both glutamatergic and cholinergic neurons in the PPN modulate slow walking, whereas CuN glutamatergic neurons were associated with escape behavior as they trigger running. Both behaviors are elicited by different cell populations that project to excitatory RSNs to produce specific gait patterns (Drew et al., 1986; Noga et al., 2003; Caggiano et al., 2018).

Dautan et al. (2021) reported that CuN glutamatergic neurons are more electrophysiologically homogeneous than PPN neurons and have mostly short-range connectivity, whereas PPN glutamatergic neurons are heterogeneous and have long-range connections in the brain. In their hands, optogenetic activation of CuN neurons elicited short-lasting muscle activation, whereas PPN neuron activation produced long-lasting increases in muscle tone associated with disrupted gait. Recently, the group of Brian Noga re-examined the distribution of locomotion-activated neurons in the brainstem of the cat using c-Fos immunohistochemistry following electrical stimulation of the MLR (Opris et al., 2019). Fos-labeled neurons were more abundant on the side of stimulation. Labeling was seen in downstream regions traditionally associated with locomotor control as well as regions associated with cardiorespiratory function. Interestingly, the study confirmed that the CuN participated extensively in the initiation of locomotion, whereas it showed little evidence for the PPT to play such a role. The results confirmed the multifaceted action of the MLR not only in the control of locomotion but in other physiological functions (for review see Ryczko and Dubuc, 2013). The multiple functions of the MLR were also recently reviewed by Noga and Whelan (2022).

Another study by the group of Dimitri Ryczko (van der Zouwen et al., 2021) revealed that the MLR is involved not only in controlling the speed of locomotion, but in steering control. Using optogenetic stimulation of the MLR in mice, they confirmed that augmenting the laser power increased the locomotor speed. On the other hand, the mice could still stop abruptly and make sharp turns during the MLR stimulation when approaching a corner in the open-field arena. The authors suggested that distinct brainstem neurons control speed and turning/stopping movements. The same group of researchers showed that selective optogenetic stimulation of glutamatergic neurons in the CuN increased the number of locomotor initiations and the time spent in locomotion (Fougère et al., 2021). Other optogenetic experiments in the rat showed that excitation of PPN cholinergic neurons causes hyperactivity in locomotion behavior, whereas inhibition of the same neurons showed hypokinesia (Xiao et al., 2016).

As indicated above, specific populations of Chx10-positive neurons have been shown to be involved in stopping locomotion when activated bilaterally (Bouvier et al., 2015). More recently, it was shown that a unilateral activation of these neurons produced an ipsilateral turn (Cregg et al., 2020). These studies suggest that the reticular formation is more important for steering than the upstream MLR. This is consistent with observations previously made in the lamprey (Deliagina et al., 2000) where the crucial role of RSNs in steering was demonstrated. Altogether, there is increasing evidence that CuN neurons modulate the speed of locomotion (Lee et al., 2014; Roseberry et al., 2016; Josset et al., 2018), whereas PPN neurons could modulate exploratory locomotion (Caggiano et al., 2018) as well as the pattern of locomotor output (Josset et al., 2018). Other optogenetic experiments in the rat showed that excitation of PPN cholinergic neurons causes hyperactivity, whereas inhibition of the same neurons caused hypokinesia (Xiao et al., 2016). Despite the significant advances made on the behavioral effects of activating or inactivating specific populations of neurons located in the different parts of the MLR, there are still discrepancies in the results. This could be due to the large physical extent of the MLR and the fact that it also contains many populations of neurons.

Optogenetic tools have also been used to characterize the ascending effects of the MLR. It was found that optogenetic activation of the PPN drives locomotion and modulates the activity of speed-modulated neurons in the cortex (Carvalho et al., 2020). Moreover, it was reported that the direct and indirect pathways in the basal ganglia had opposing effects on locomotor modulation (Bateup et al., 2010; Kravitz et al., 2010) and that the basal ganglia regulated the MLR with reciprocal inputs from the latter region (Roseberry et al., 2016). Three neurochemically distinct cell types within the MLR were examined: glutamatergic, GABAergic, and cholinergic neurons. The glutamatergic population was found to encode locomotor state and speed. The activation of GABAergic neurons in the MLR caused the locomotion to stop, either through local effects on glutamatergic neurons or downstream effects to the reticular formation. The authors showed that cholinergic neurons within the MLR were insufficient to elicit locomotion, but they could modulate ongoing locomotion. An important contribution of dopaminergic inputs on MLR neurons was recently described in the mouse (Sharma et al., 2018) as seen in lampreys (Ryczko et al., 2013, 2016b,^2017^; Ryczko and Dubuc, 2017). Altogether, these studies highlight the power of up-to-date genetic tools in defining the contribution of specific cell populations on behavior. The detailed connectivity and neural activity within these locomotor networks remain to be defined more precisely. To fully understand the role of the MLR in the control of locomotion, it is necessary not only to characterize its downstream projections to the reticular formation, but also its inputs from the forebrain and other brainstem structures. This topic, although highly important, exceeds the scope of this review.

## 5. Conclusion

In this review, we have presented a general overview of some of the brainstem mechanisms involved in the control of locomotion. We argue that results obtained in more basal vertebrates such as the lamprey, zebrafish, and salamander, can provide useful cues to orient further research in mammals. As indicated above, the recent development of genetic tools has significantly revived enthusiasm on the supraspinal control of locomotion in mammals. Optogenetic tools have been very useful for linking neuronal activation or inactivation and the behavioral output, as well as to highlight the specific contributions of different neurotransmitter systems. On the other hand, to fully identify the underlying cellular mechanisms, synaptic connectivity should be known. For instance, the connectivity between different populations of neurons in the mammalian MLR and the downstream RSNs must be defined in details. On that matter, other animal models could provide new important information. Genetic tools have been efficiently used in zebrafish in combination with imaging and electrophysiological techniques to successfully study synaptic connectivity (Severi et al., 2014, 2018; Thiele et al., 2014; Thouvenin and Wyart, 2017; Carbo-Tano et al., 2022). The activity of entire populations of neurons can be monitored and characterized during ongoing locomotion. Classic electrophysiological approaches can be used to define the synaptic connectivity between neurons involved in the control of locomotion. Moreover, whole populations of genetically identified neurons can be selectively activated or inactivated, bridging the gap between cellular mechanisms and behavior. It is likely that the zebrafish model will be increasingly useful to characterize the cellular mechanisms underlying locomotion.

## Author contributions

PL-O and RD were involved in the conception of this review article, the writing and revision of the text, and the conception of the figures. Both authors approved the submitted version.
